# Effects of controlled breathing exercises and respiratory muscle training in people with chronic obstructive pulmonary disease: results from evaluating the quality of evidence in systematic reviews

**DOI:** 10.1186/1471-2466-14-184

**Published:** 2014-11-21

**Authors:** Christine Råheim Borge, Kåre Birger Hagen, Anne Marit Mengshoel, Ernst Omenaas, Torbjørn Moum, Astrid Klopstad Wahl

**Affiliations:** Department of Health Sciences, University of Oslo, Oslo, Norway; Department of Medicine, Lovisenberg Diaconal Hospital, Oslo, Norway; Department of Rheumatology, National Resource Centre for Rehabilitation in Rheumatology, Diakonhjemmet, Oslo, Norway; Centre for Clinical Research, Haukeland University Hospital, Bergen, Norway; Department of Behavioral Sciences in Medicine, University of Oslo, Oslo, Norway

**Keywords:** Chronic obstructive Pulmonary disease, Controlled breathing, Respiratory muscle training, Diaphragmatic breathing, Pursed lip breathing, Overview

## Abstract

**Background:**

This paper reviews evidence and quality of Systematic Reviews (SRs) on the effects of breathing control exercises (BCEs) and respiratory muscle training (RMT) on breathlessness/dyspnea and other symptoms, and quality of life (QOL) for individuals with chronic obstructive pulmonary disease (COPD).

**Methods:**

A search for BCE and RMT literature in COPD published between January 1, 2002 and December 31, 2013 was performed in the following databases: PubMed, Ovid, CINAHL, PsycINFO, AMED, Cochrane and PEDro. The AMSTAR criteria were used to evaluate quality.

**Results:**

After reviewing 642 reports, seven SRs were identified on RMT and BCEs. Three SRs were of high quality, three were of moderate quality, and one was of low quality. Two high-quality SRs reported significantly beneficial effects of RMT on dyspnea, and one reported significant effects on disease-specific QOL and fatigue. In these SRs, pooled data analyses were performed with three to fourteen single randomised control trials (RCTs) included in the analysis. In one of the SRs the quality of the single RCTs were rated by the authors to be between 5–7 (with10 best) and in the other one the quality of the single RCTs were rated to be between 30-83% of the maximum score.

One high-quality SR found a significant positive effect of BCE based on pooled data analysis with two single RCTs in regard to pursed-lip breathing (PLB) on breathlessness. In this SR, one single RCT on diaphragmatic breathing (DB) and another one on yoga breathing (YB) showed effect on disease-specific QOL. The single RCTs included in the SR were rated by the authors in the SRs to be of low and moderate quality.

**Conclusions:**

Based on three high-quality SRs performing pooled data analyses, there is evidence that RMT has effect on breathlessness, fatigue and disease-specific QOL and PLB on breathlessness. There is also evidence that single studies on DB and YB has effect on disease-specific QOL. Few RCTs are available and the variable quality of the single RCTs in the SRs, seem to require more RCTs in particular for BCEs, but also RMT before conclusions regarding effects and high quality SRs can be written.

**Electronic supplementary material:**

The online version of this article (doi:10.1186/1471-2466-14-184) contains supplementary material, which is available to authorized users.

## Background

Chronic Obstructive Pulmonary Disease (COPD) is characterised by airflow limitation due to obstruction of airways. Due to peripheral airway obstruction, air volume may become trapped in the lungs (i.e. hyperinflation) [1]. The respiratory rate may increase because of inspiration, which is initiated before emptying the lungs of air. Adjustment of rapid shallow breathing may lead to respiratory muscles fatigue. Hyperinflation may lower the dome of the diaphragm, shorten respiratory muscle fibers, and impair the possibility of muscle contraction. In addition, gas exchange may be inefficient. Hence, patients with COPD might develop symptoms of breathlessness or dyspnea [1–3]. Previous research has found that breathlessness is associated with symptoms of depression, anxiety, fatigue, sleeping difficulties, pain [4], and reduced quality of life (QOL) [5]. The main goal of management and treatment in COPD is to improve symptoms and QOL [1].

**Figure 1:**
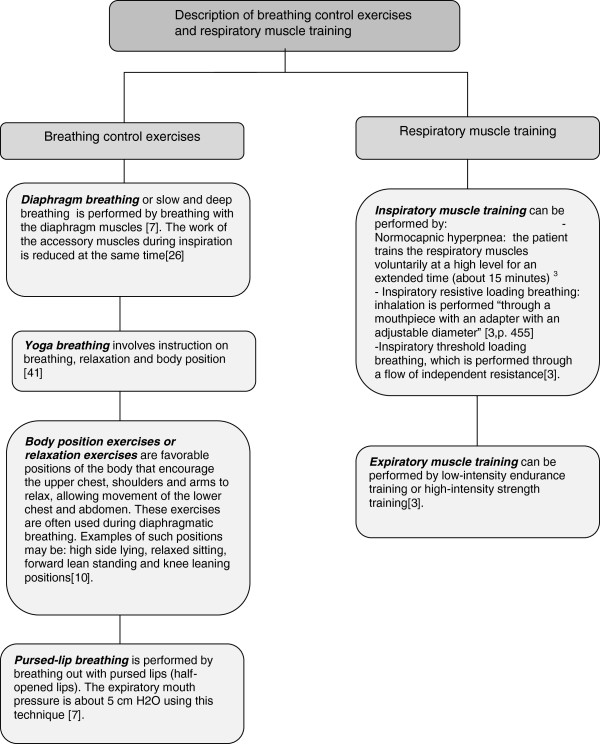
**Description of breathing control exercises and respiratory muscle training.**

Various breathing control exercises (BCEs) and respiratory muscle training (RMT) are being used [6–10] to improve breathlessness. For example, BCEs include diaphragmatic breathing (DB), pursed-lip breathing (PLB), relaxation techniques (RT), and body position exercises (BPEs). BCEs aim to decrease the effort required for breathing and assist relaxation by deeper breathing, which may result in an improved breathing pattern through decreased respiratory rate and reduced breathlessness [3, 6, 10]. Figure 1 provides a description of the differences for each exercise and training performance. In regard to RMT, the aim is to improve muscle strength and endurance where the respiratory muscles are impaired, hopefully resulting in greater effort to control breathing pattern and reduce breathlessness [9]. RMT requires a training program using an adjusted breathing resistance device [3, 10]. (See Figure 1 for further information.)

The practice of evidence-based medicine and health care are often based on the results of knowledge extracted from systematic reviews [11, 12]. A systematic review is “a form of literature review that requires a documented search strategy and explicit inclusion and exclusion criteria for studies reviewed, reducing author bias toward or against particular methods or outcomes” [11], p. 483. Systematic reviews (SRs) report various beneficial improvements on BCEs and RMT [13, 14]. However, not all SRs are well conducted, e.g. not following the advice and criteria on how to prepare an SR [11], and biased knowledge may be a consequence.

Although BCEs and RMT are often mentioned in the literature as common exercises and training for non-pharmacological treatments used for the management and improvement of breathlessness in COPD [6–10], the aims and the mechanisms of the two strategies are different. These differences have been discussed to a limited degree in previous literature; however, including articles that evaluate BCEs and RMT in the same review may clarify these differences.

Therefore, in order to better understand and appreciate the evidence regarding the effects of BCEs and RMT on subjective factors (such as symptoms and quality of life) in the context of COPD, it is valuable to evaluate the quality of the SRs` examining the effect of these exercises and training procedures. Such an overview may reveal areas lacking high-quality studies and identify specific BCEs and RMT skills that might be of special benefit to patients with COPD. Our aim of the present paper is to summarise the results from SRs that evaluate the effect of breathing control exercises and respiratory muscle training on breathlessness, other symptoms, and quality of life in patients with COPD, taking into account the quality of the SRs.

## Method

### Overviews of reviews

We conducted an overview of SRs, including both Cochrane SRs and non-Cochrane SRs [15], to summarize the evidence on the effects of breathing control exercises and respiratory muscle training.

### Inclusion and exclusion criteria

We included articles defined as SRs of the authors of the BCEs and RMT most commonly used and referred to in the literature [6–10]. These where; respiratory muscle training and BCEs of slow and deep breathing, diaphragmatic breathing, pursed lip breathing, relaxation techniques and body position exercises in humans evaluating the effects on breathlessness or dyspnea, other symptoms and quality of life in adult patients (>18 years) at all stages of COPD. We included SRs based on systematic literature search [11] in databases such as MEDLINE. We therefore included reviews that had performed a search in at least one database, along with SRs in all languages. The last written and updated SR of a previous SR and SRs that met at least one of the Measurement Tool to Assess Systematic Reviews (AMSTAR) criteria [16] were considered eligible.

We excluded guidelines and lists of general management advice; other diseases than COPD; narrative reports (i.e. aiming to focus on an understanding of a concept) [11]; reviews titled as literature reviews or integrative reviews, evaluations of ventilator support (such as noninvasive ventilation); only evaluations of pulmonary rehabilitation/self-management courses in which a breathing control exercise was one of several treatments; evaluations of general muscle training and cardiovascular exercise programs, medication interventions, and mini-pep or active sputum mobilization; explanations of how the respiratory system works; not evaluating the outcomes on breathlessness, other symptoms and quality of life; and SRs that did not meet any of the AMSTAR criteria.

### Outcome measures

*Breathlessness* and *dyspnea* are concepts that often are used interchangeably. Although they have been described as two distinct concepts [17], in this paper, we used them in the sense of a “subjective experience of breathing discomfort that is comprised of qualitatively distinct sensations that vary in intensity” [18], p.401, [19], p. 322. We considered SRs which included studies using measures of breathlessness or dyspnea as outcome variables.

*Symptoms* are defined as “subjective experience reflecting changes in the biopsychosocial functioning, sensations, or cognition of an individual” [20]. In this overview, “symptoms” include those relating to diseases such as dyspnea, and those that could be a consequence of the disease or of other diseases. Such symptoms could for instance be fatigue, depression or anxiety. We considered SRs that included instruments measuring symptoms.

*Quality of life (QOL)* is a concept involving several aspects of a subject’s experience of life, including global QOL, health related QOL and disease-specific QOL [21]. In this overview of SRs, we have focused on instruments involving disease related QOL and health related QOL.

### Search strategy

Our search was performed in “PubMed”, “Ovid”, “CINAHL”, “PsycINFO”, “AMED”, “Cochrane” and “PEDro” using the following Medical Subject Headings (MeSh) terms or key words/concepts: 1) “Lung disease”, 2) “Lung disease, obstructive”, 3) “Pulmonary disease, chronic obstructive”, 4) “Pulmonary emphysema”, 5) “Bronchitis chronic”, 6) “Pharmacology”, 7) “Oxygen therapy”, 8) “Respiration”, 9) “Breathing exercises”, 10) “Yoga”, 11) “Pranayama”, 12) “Mind–body therapies”,13) “Muscle stretching exercises”, 14) “Relaxation” and 15) “Breathing control exercises”, 16) “Diaphragmatic breathing” and 17) “Pursed lip breathing”. Additional categories were: 18) combinations of search terms 1–5 using “OR”, 19) combinations of search terms 6–7 using “NOT” (searched in PubMed only) and 30) combinations of search terms 20–29 using “OR”.’

When possible we used the limitations “human,” “review” and publications from January 1, 2002 to December 31, 2013 in the databases. For further details of the search, see Additional file 1.

### Data collection and analyses

We used the Measurement Tool to Assess Systematic Reviews (AMSTAR) [16] method to evaluate the methodological quality of the different papers. The AMSTAR criteria are listed in Table 1. The 11 criteria of the AMSTAR were rated as “yes” (criteria were met), “no” (criteria were not met), “cannot answer” (unclear information) or “not applicable” (criteria could not be evaluated because of the design of background studies in the reviews). Ratings of “yes” were scored 1, and ratings of “no,” “cannot answer” and “not applicable” were scored 0. The highest possible AMSTAR score is 11. Review quality was classified by AMSTAR score: 0–4 = low quality, 5–8 = moderate quality and 9–11 = high quality [22].

**Table 1:** **AMSTAR score in the different reviews**

AMSTAR criteria	Holland et al. 2012[14]	Roberts et al. 2009[30]	Geddes et al. 2008[13]	Gosselink et al. 2011[31]	O’Brian et al. 2008[32]	Shoemaker et al. 2009[34]	Thomas et al. 2010[33]
**1.** Was an “a priori” design provided?	Yes	Yes	Yes	Yes	Yes	Yes	Yes
**2.** Was there duplicate study selection and data extraction?	Yes	Ca	Yes	Yes	Yes	Ca	Yes
**3.** Was a comprehensive literature search performed?	Yes	Yes	Yes	Yes	Yes	Yes	Yes
**4.** Was the status of publication used as an inclusion criterion? (i.e. language, grey literature)	Yes	No	No	Yes	No	No	No
**5.** Was a list of studies (included and excluded) provided?	Yes	No	No	Yes	No	No	Yes
**6.** Were the characteristics of the included studies provided?	Yes	No	Yes	Yes	Yes	Yes	Yes
**7.** Was the scientific quality of the included studies assessed and documented?	Yes	Yes	Yes	Yes	Yes	Yes	Yes
**8.** Was the scientific quality of the included studies used appropriately in formulating conclusions?	Yes	Yes	No	No	No	Yes	Yes
**9.** Were the methods used to combine the findings of studies appropriate?	Yes	Na	Yes	Yes	Yes	No	Yes
**10.** Was the likelihood of publication bias assessed?	Yes	Na	No	Yes	Ca	No	Yes
**11.** Were potential conflicts of interest included?	Yes	No	Yes	Yes	No	No	No
**Sum AMSTAR**	11	4	7	10	6	5	9
**Quality**	High	Low	Moderate	High	Moderate	Moderate	High

Yes = 1, No = 0, Cannot answer (Ca) = 0, Not applicable (Na) = 0.

AMSTAR score of 0–4 = low quality, AMSTAR score of 5–8 = moderate quality, AMSTAR score of 9–11 = high quality.

In order to identify SRs, one reviewer (CRB) extracted data regarding population, interventions, comparisons and outcomes (inclusion criteria) and evaluated the methodological quality of the included trials. After duplicated articles had been removed, the search was first evaluated based on their title. Potential abstract were then assessed for eligibility. After excluding articles that were not eligible full articles were read for further details. A second reviewer (AKW) independently verified the accuracy of numeric results and evaluated the methodological quality. The search strategy and findings were discussed with a third reviewer (KBH). Finally, three other co-authors (EO, AMM and TM) were brought in to discuss the method and the results together with CRB, AKW and KBH.

### Data synthesis

The included SRs are organized and presented in three tables. The data from the SRs were directly extracted. Table 2 presents descriptive information of the SRs; author, year, aim and search parameters. Table 3 categorizes the effects found by the SRs; pooled statistics of main effect variable, intervention description and authors conclusions about the different breathing control exercises and respiratory muscle training.. Evaluations of quality of the SRs, according to the AMSTAR criteria, are presented in Table 1.

**Table 2:** **Descriptive information of included systematic reviews**

Author and year	Aim, sources of electronic literature search (year) and language of search
**Diaphragmatic breathing, pursed-lip breathing and yoga breathing**	
Holland et al. (2012) [14]	*Aim:* “1. To determine whether breathing exercises in people with COPD have beneficial effects on dyspnoea, exercise capacity and health-related quality of life compared with no breathing exercises in people with COPD. 2. To determine whether there are any adverse effects of breathing exercises in people with COPD.”
	*Search:* Cochrane Central Register of Controlled Trials (CENTRAL) MEDLINE, EMBASE, CINAHL, AMED and PsycINFO, and hand searching of respiratory journals and meeting abstracts (Years: from inception up to October 2011)
	*Language:* No language restriction
	*Participants:* COPD patients in stable condition (mean FEV _1_% : 30%-51%, mean age: 51–73 years)
**Pursed-lip breathing**	
Roberts et al. (2009) [30]	*Aim*: “Determine the evidence for teaching pursed lips breathing (PLB) to patients with stable chronic osbructive pulmonary disease (COPD)”
	*Search:* MEDLINE, PEDro and CINAHL (Years: NR)
	*Language:* English
	*Participants:* Stable COPD*
**Respiratory muscle training**	
Geddes et al. (2008) [13]	*Aim:* “To determine the effect of inspiratory muscle training (IMT) on inspiratory muscle strength and endurance, exercise capacity, dyspnea and quality of life in adults with chronic obstructive pulmonary disease (COPD).”
	*Search:* Cochrane Collaboration Methods, MEDLINE, CINHAHL, EMBASE (Years: inception to January 2007)
	*Language:* English
	*Participants:* COPD patients (mean FEV _1_% : 24%-52%, mean age: 62–68 years)
Gosselink et al. (2011) [31]	*Aim:* “1. Investigate the effects of IMT as stand-alone therapy or added to general exercise training. 2. Identify patient characteristics associated with favourable effects of IMT and 3. Identify the most appropriate training modality in terms of strength or endurance training for IMT.”
	*Search:* Medline, CINAHL, Cochrane Central Register of Controlled Trials, EMBASE, PEDro,
	DocOnline, ATS and ERS congress (Years: 2000–2008)
	*Language:* No language restriction
	*Participants:* COPD patients (mean FEV _1_% :30%-55%, mean age:55–73 years)
O’Brian et al. (2008) [32]	*Aim:* “To determine the effect of inspiratory muscle training (IMT) (alone or combined with exercise and/or pulmonary rehabilitation) and compare with other rehabilitation interventions among adults with chronic obstructive pulmonary disease (COPD)”
	*Search:* Cochrane Collaboration Methods (Years: up to December 2005)
	*Language:* English
	*Participants:* COPD patients (mean FEV _1_%_:_ less than 65%, mean age: 56–72 years)
Shoemaker et al. (2009) [34]	*Aim:* “To interpret the literature and assess the quality of evidence regarding the clinical benefits of IMT and the application of this evidence and its limitations to clinical practice by reviewing studies that used training intensity-controlled IMT compared with sham or no intervention.”
	*Search:* CINAHL, PubMed, Medline and ProQuest (Years: no limitation)
	*Language:* English
	*Participants:* COPD patients (mean FEV _1_% : 33%-55%, mean age: 41–71 years)
Thomas et al. (2010) [33]	*Aim:* “Determine the impact of home-based physiotherapy interventions on breathlessness during activities of daily living (ADL) in severe chronic obstructive pulmonary disease (COPD)”.
	*Search:* AMED, CINAHL, Cochrane Central Register of Controlled Trials, Embase, Medline and Physiotherapy Evidence Database (PEDro) (Years: inception to week 20, 2008), Reference lists of the latest official statements of the America Thoracic Society, the Thoracic Society of Australia and New Zealand, the British Thoracic Society, the European Respiratory Society and GOLD.
	*Language:* English
	*Participants:* Severe COPD (mean FEV _1_%: ≤50%, above 18 years)

**NR:** Not registered, *Severity and/or age not registered, FEV1%: predicted forced expiratory volume in one second.

**Table 3:** **Outcome measures and main effects in the included SR’s**

Authors (year)	BCE or RMT	I and C group (design of comparison)	Intervention descriptor and duration	Outcome variable (measure)	No. of trials (No. of subjects)	Pooled statistics on main effect variables MD/WMD/NU and/or SES (95%CI), p-value, (Effect in favor of intervention or control)	Authors’ comments on quality measure	Authors’ conclusion
Holland et al. (2012) [14]	DB, PLB, YB	PLB compared with no BE (RCT)	8 -12 weeks training	Dyspnea (BS)	2 (19)	NQPD (NS)	Low QOE*	The effects of breathing exercises on breathlessness and well-being were variable.
				Dyspnea (UCSD Shortness of Breath Questionnaire)	2 (19)	NQPD (NS)	Low QOE*	
				Dyspnea (MRCS)	1 (30)	NQPD (I)	NR	
				Dyspnea (Hiratsuka Scale)	2 (60)	MD -12.94 (-22.29, -3.60), p = 0.0066, (I)	Low QOE*	
				Health condition (Hiratsuka Scale)	2 (60)	MD 6.19 (-5.24,17.61), p = 0.29 (NS)	NR	
				Mood (Hiratsuka Scale)	2 (60)	MD 1.08 (-9.60,11.75), p = 0.84 (NS)	NR	
				Social function (Hiratsuka Scale)	2 (60)	MD 11.69 (-0.91,24.28), p = 0.069 (NS)	NR	
				House work (Hiratsuka Scale)	2 (60)	MD 15.58 (0.5,30.66), p = 0.043 (C)	NR	
				Headache (Hiratsuka Scale)	2 (60)	MD -3.30 (-12.37,5.77), p = 0.48 (NS)	NR	
				Appetite (Hiratsuka Scale)	2 (60)	MD 8.42 (-5.3,22.15), p = 0.23 (NS)	NR	
				Well being (Hiratsuka Scale)	2 (60)	MD 0.09 (-9.80,9.98), p = 0.99 (NS)	NR	
				QOL (Cai scale)	1 (89)	NQPD (I)	NR	
		DB compared with no BE (RCT)	4-12 weeks training	Dyspnea (MRCS)	1(30)	NQPD (NS)	Moderate QOE*	
				QOL (St. George RQ)	1(30)	NQPD (I)	Moderate QOE*	
		Yoga compared with no BE (RCT)	12 weeks training	Dyspnea intensity (BS)	1 (29)	NQPD (NS)	Low QOE*	
				Dyspnea distress (BS)	1 (29)	NQPD (NS)	Low QOE*	
				Dyspnoea-related QOL	1 (29)	NQPD (NS)	NR	
				Health QOL (St. George RQ)	1 (45)	NQPD (I)	Moderate QOE*	
		PLB compared with EMT (RCT)	4-12 weeks training	Dyspnea (BS, SOBQ)	2 (17)	NQPD (I (PLB) on 12 weeks, NS on 4 weeks)	Low QOE*	
							Low QOE*	
				Dypsnea (UCSD Shortness of Breath Questionnaire)	2 (17)	NQPD (NS)		
		DB, PLB and nutritional (RCT)	NR	QOL (Cai scale)	1 (71)	NQPD (NS)	NR	
Roberts. et al. (2009) [30]	PLB	PLB during everyday activities or during exercise (PP)	NR in table #	Dyspnea (BS)	5 (110)	40% relief (range 0%–to 63%) # (NR)	Low and moderate QOE # **	PLB has a role in the symptomatic management of stable COPD.
Geddes et al. (2008) [13]	IMT	Inspiratory muscle training versus intervention sham (RCT)	Intensity ≥30%–60% or max load, Pimax (threshold) 15–30 minutes 1–2 pr. day, 3–7 days pr. week for 5–24 weeks.	Dyspnea (BS)	4 (99)	WMD -1.76, (-2.35, -1.16),	No score given. Descriptive summary of the MQ is provided.	IMT improves measure of quality of life and decreases dyspnea for adults with stable COPD.
						p <0.00001, (I)		
				Dyspnea (TDI focal score)	5 (96)	WMD 2.55, (0.92, 4.19), p = 0.002, (I)		
			60% MVV (normocapnic hyperpnea tube breathing) 15 minutes × 2 pr. day, 7 days a week for 5 weeks.	Dyspnea (TDI functional impairment)	3 (56)	WMD 0.72, (0.14, 1.31), p = 0.02, (I)		
				Dyspnea (TDI magnitude of task)	3 (56)	WMD 0.74, (0.49, 1.0), p <0.00001, (I)		
				Dyspnea (TDI magnitude of effort)	3 (56)	WMD 0.48, (0.24, 0.72), p <0.0001, (I)		
				Quality of life (CRQ total score)	2 (69)	WMD 0.33, (0.19, 0.47), p <0.00001, (I)		
Gosselink et al. (2011) [31]	IMT	IMT versus control (RCT)	Intensity ≥30%, Pimax (threshold load) or endurance training in controlled manner (inclusion criteria), 15–90 minute × 2–3 pr. day, 5–7 days a week, for 4 weeks to 12 months.##	Dyspnea (BS)	14(NR)	NU -0.9, SES -0.45, (-0.66 to -0.24),	MQ score from 30–83% (median 59%) of the maximum score.**	IMT improves dyspnea and health QOL.
						p <0.001, (I)		
				Dyspnea (TDI score)	4 (NR)	NU +2.8, SES 1.58, (0.86–2.3),		
						p <0.001, (I)		
				Dyspnea (CRQ)	9 (NR)	NU +1.1, SES 0.34, (-0.03–0.71),		
						p = 0.068, (I)		
				Quality of life (CRQ score)	9 (NR)	NU +3.8, SES 0.34, (0.09–0.6), p = 0.007, (I)		
				Fatigue (CRQ score)	10 (NR)	NU + 0.9, SES 0.27, (0.03–0.5),		
						p = 0.024, (I)		
				Emotion (CRQ score)	10 (NR)	NU + 0.5, SES 0.19, (-0.04–0.42),		
						p = 0.107		
				Mastery (CRQ score)	10 (NR)	NU–0.005, SES 0.09, (-0.14–0.33), p = 0.432		
O’Brien et al. (2008) [32]	IMT	Combined IMT and exercise versus exercise alone (RCT)	Intensity 30%, Pimax-60% (threshold), 30 minutes × 1 pr. day 5 days a week for 16 weeks. <72% MVV (Normocapnic hyperventilation) 1–20 minutes × 1 pr. day, 3 days a week for 8 weeks.	Quality of life CRQ dyspnea	2 (57)	WMD -1.94, (-2.88, -1.01), p <0.0001, (I)	No score given. MQ information given in a table in the SR.	Results of dyspnea and QOL are less clear. Further trials are required.
				Quality of life CRQ fatigue	2 (57)	WMD -0.23, (-3.85, 3.4), p = 0.9		
Shoemaker et. al. (2009) [34]	IMT	IMT versus control	Intensity load 17-100% Pimax, 15–30 minutes daily, 3–7 days a week for 8–24 weeks	Dyspnea (during IMT)	3 (85)	NQPD ##	Score 40-90% level 1b ****	IMT improve dyspnea and QOL in COPD patients
				QOL	6 (188)	NQPD ##		
Thomas et al. (2010) [33]	IMT	IMT at home versus control (RCT)	No information on intensity, 30–60 minutes pr. day × 3–6 pr. week, for 3–12 months.	Dyspnea (TDI score)	3 (57)	MD 2.36 (0.76, 3.96), p = 0.004, (I) ##	Score of 5 in one study and 7 in two studies.***	IMT may improve breathlessness during activities of daily living in severe COPD.

**BCE:** Breathing control exercise, **BS:** Borg Score (range 0–10), **C:** Control, **CI:** Confidence interval, **CRQ:** Chronic Respiratory Questionnaire (range 0–7)**, DB:** Diaphragmatic breathing, **EMT:** Expiratory muscle training, **I:** Intervention, **IMT:** Inspiratory muscle training**, MD:** Mean difference, **MRCS:** Medical Research Council Score, **MQ:** Methodological quality, **N**: Number, **NQPD** = No quantitative pooling data, **NR:** Not registered, **NS**: Not significant, **NU**: Natural units, **PLB:** Pursed-lip breathing, **QOE**: Quality of evidence, **RCT:** Randomized controlled trial, **RQ:** Respiratory Questionnaire, **SES:** Summary effect size, **SOBS:** Shortness of Breath Score, **St. George RQ:** St. George Respiratory Questionnaire (range 0–100), **TDI**: Transition Dyspnea Index (range -9–9), **UCSD Shortness of Breath Questionnaire:** University of California San Diego Shortness of Breath Questionnaire (range 0–120), **WMD:** Weighted mean difference, **YB:** Yoga breathing.

*Used the GRADE Working grades of evidence (High quality: further research is very unlikely to change our confidence in the estimate of effect; Moderate quality: Further research is likely to have an important impact on our confidence in the estimate of effect and may change the estimate; Low quality: Further research is very likely to have an important impact on our confidence in the estimate of effect and not likely to change the estimate).

**Used a modification of the framework for methodological quality used by Smith et al.

***Used PEDro methodological quality score (range 0–10). Higher scores indicate better quality.

**** Used the evaluation scale developed by Medlicott and Harris. Scores: 80–100% = strong quality; 60–79% = moderate quality; <59 = low quality. Studies with weak methodological rigor were assigned an evidence level of 2b.

#Difficulty reading information from tables and text.

##Single studies not reported due to difficulties of reading out the exact design and effect from text and/or tables.

## Results

### Selection of SRs

**Figure 2:**
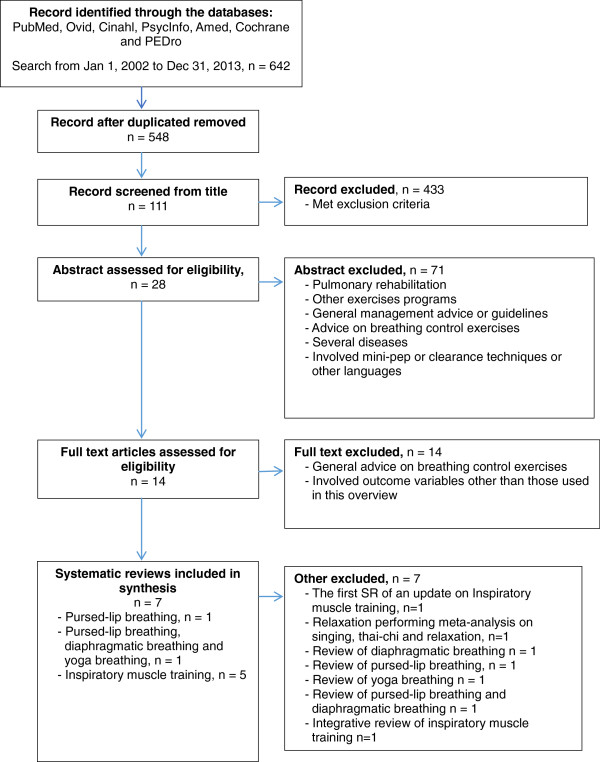
**Flow chart of inclusion and exclusion process.**

Figure 2 shows the SR inclusion process. The literature search identified 642 reviews. After checking titles and abstracts against the inclusion and exclusion criteria, we finally included seven relevant SRs on breathing control exercises. We found one SR evaluating relaxation exercises [23]. This SR was based on complex intervention on depression and anxiety and the authors had pooled their data on three different exercises (i.e. relaxation singing, yoga, thai-chi). In a sub-group analysis no effect was found on symptoms of depression and anxiety. Neither singing or thai-chi exercises were included as exercises in the present overview and we therefore excluded this SR. Further, we excluded one SR that was a previous version of an update [24]. We also excluded five reviews that had performed literature searches, but where the authors had defined their articles as integrative reviews or literature reviews [25–29]. These were therefore not considered to be SRs.

### Description of Included SRs

Table 2 shows the included SRs. With respect to BCE; one on pursed-lip breathing [30] and one SR on diaphragmatic breathing, pursed-lip breathing and yoga breathing [14] were included. Regarding RMT five SRs on inspiratory muscle training [13, 31–34] were identified. There were no SR evaluations of the effects on breathlessness/dyspnea, other symptoms or QOL, on relaxation techniques, body position exercises or expiratory muscle training in independent SRs of people with COPD during the search period. For further information, see Table 2.

### Effect and methodological quality of included SRs

The effects on breathlessness/dyspnea, other symptoms and QOL are presented in Table 3, and the results of the quality assessment are presented in Table 1.

### Respiratory muscle training

According to the AMSTAR criteria, the SRs of Gosselink et al. [31] and Thomas et al. [33] were of high quality (see Table 1 for details). Gosselink et al. [31] pooled data from 14 Randomized Control Trials (RCTs) comparing those who received inspiratory muscle training with a control group, and they found a significant effect (p <0.001) in favour of inspiratory muscle training on dyspnea, with a summary effect size of -0.45 (95% CI -0.66 to -0.24), corresponding to -0.9 on the Borg Scale, which ranges from 0 to 10 (Table 3). For the four RCTs using the Transition Dyspnea Index (range -9 to 9), a significant effect (p ≤0.001) of inspiratory muscle training of 2.8 was found, corresponding to a summary effect size of 1.58 (95% CI 0.86–2.3) (Table 3). When data from nine RCTs were pooled, a significant summary effect of 3.8 (p <0.007) on disease-specific QOL measured by the Chronic Respiratory Questionnaire (CRQ) (range 0 to 7) was found, with a summary effect size of 0.34 (95% CI 0.09 to 0.6) (Table 3). For other symptoms, statistical pooling of ten RCTs revealed a significant effect (p = 0.024) on fatigue, with a summary effect size of 0.27 (95% CI 0.03–0.5) but no significant effect on emotion and mastery. All effects were in favour of inspiratory muscle training. The methodological quality was rated by the authors in the SR to range from 30–83% of the maximum score (median of 59%).

Thomas et al. [33] pooled data from three RCTs that compared those who received respiratory muscle training (inspiratory muscle training and expiratory muscle training) at home with controls, and they found a significant effect (p = 0.004) on dyspnea with a mean difference of 2.36 (95% CI 0.76–3.96) on the Baseline and Transition Dyspnea Indexes( BDI/TDI) score (range -9 to +9). The PEDro score rated by the authors in the SR showed a methodological quality of 5 in one study and 7 in two studies (range 0–10, where10 is best quality).

The SRs of Geddes et al. [13], O’Brien et al. [32] and Shoemaker et al. [34] were of moderate quality according to the AMSTAR criteria (details provided in Table 1). Geddes et al. [13] investigated the effect of inspiratory muscle training versus sham intervention, and concluded that inspiratory muscle training may improve QOL and decrease dyspnea in stable COPD. O’Brien et al. [32] investigated the effect of inspiratory muscle training versus exercise or a combination of exercises and inspiratory muscle training, and they concluded that the effect on dyspnea and QOL was unclear. Shoemaker et al. [34] did not perform any statistical pooling but concluded that inspiratory muscle training improved dyspnea and QOL in COPD patients, based on moderate- to high-quality trials included in the SR.

No SRs were found on the use of expiratory muscle training alone.

### Breathing control exercises

Holland et al. [14] performed an SR on diaphragmatic breathing, pursed-lip breathing and yoga breathing, which was assessed as being of high methodological quality. They performed eight pooled data analyses, including two RCTs in the pooled data analysis that compared pursed-lip breathing with no breathing practice. A significant effect (p = 0.0066) was only found on dyspnea in favour of pursed-lip breathing, with a mean difference of -12.94 (95% CI -22.29 to -3.60) on the Hiratsuka Scale, which ranges from 0 to 100 (Table 3). Further, regarding single trials, one showed an effect on shortness of breath and one on QOL when comparing pursed lip breathing with no BCEs. The evidence of single trials performed on pursed lip breathing was rated by the authors in the SR to be of low quality. Two trials on diaphragmatic breathing and four on yoga breathing showed effects only in one study in each exercise on disease related QOL when comparing with no BCEs. The evidence of single RCTs performed on diaphragmatic breathing rated by the authors in the SR to be of moderate quality and on yoga breathing of low to moderate quality. According to the authors one study yielded a small effect on pursed lip breathing compared with expiratory muscle training after a 12 weeks intervention. The authors concluded that breathing exercises on breathlessness and wellbeing showed variable effects.

Roberts et al. [30] performed a review on pursed-lip breathing and included two RCTs and nine studies with pre–post design. They concluded that 40% of dyspnea was relieved when pursed-lip breathing was used [30]. The evidence in the included studies was rated to be of low to moderate quality.

For further information, see Tables 3 and 1.

## Discussion

We reviewed the quality of SRs on the effects of BCEs and RMT on breathlessness/dyspnea, other symptoms, and QOL in patients with COPD. Our main result shows that, in regard to inspiratory muscle training, two high-quality SRs have reported significant effects on the relief of dyspnea [31, 33] and fatigue, and improved QOL [31]. In addition, among eight pooled data analyses on pursed-lip breathing, only one analysis showed a positive effect on the reduction of dyspnea in a high-quality SR. In addition, significant effects on disease-specific QOL were found in one single study of diaphragmatic breathing and one single study of yoga breathing. The results in the SRs of BCEs and RMT were based on single RCTs of variable quality.

### Discussion of the methodological challenges in the SRs

There are several methodological challenges both in the single studies included in the SRs and in the SRs on BCEs and respiratory RMT. Therefore, care should be taken when interpreting the findings in low- and moderate-quality SRs as well as in high-quality SRs.

Some of the methodological challenges in the SRs are related to low scores on the AMSTAR criteria (Table 1) (i.e. criteria numbers 4, 8, 9, 10), but also on the quality of the included single studies in the SRs.

Apart from two SRs [14, 31] (i.e. high quality) using no language restriction, the rest used only English-language articles in their reviews (i.e. Table 2) [13, 30, 32–34]. It has been found that journals using the English language tend to report interventions with positive results, whereas journals published in other languages also include studies with negative results [35]. There was also limited information in the SRs with regard to including grey literature (i.e. abstracts from international congresses, unpublished work, book chapters, and theses). The exclusion of grey literature has been found to increase intervention effects in meta-analyses [36]. However, we cannot be certain that results would have been different in the included SRs even if they had not included restrictions regarding languages or forms of literature, and therefore, a potential publication bias of under-reporting on lacking effects cannot be ruled out.

Being able to include RCT design gives advantages of scoring higher on the AMSTAR criteria. The small number of RCT studies available are therefore one of the reasons for the low-quality score according to the AMSTAR criteria found in the SR by Roberts et al. [30] on pursed-lip breathing. In contrast, Holland et al. [14] scored high on the AMSTAR criteria, due in part to their performing pooled analysis in one of the exercises (i.e. pursed-lip breathing). However, in the same SR, few trials were available on diaphragmatic breathing and yoga breathing. This is not captured in the AMSTAR score. Although prospective pre- and post-design may be supplemental in studies, RCT design has been determined to be the ideal way of measuring intervention effects [37]. RCT design will, in turn, provide the possibility to do pooled data analyses.

Although the AMSTAR criteria give higher quality in SRs on the background of including RCT designs, the quality of single RCTs included in the SRs is essential for the quality of the data material undergoing analysis. This is shown in the SR by Holland et. al. [14] where they performed eight pooled data analyses on pursed-lip breathing, but the two single trials included had low-quality evidence, and only one of the analyses showed effects on dyspnea. In Thomas et. al. [33] SR of inspiratory muscle training, the single studes included in the pooled analyses were mostly rated and interpreted to be of moderate to high quality and the author concluded that further research was needed. Gosselink et. al. [31], assessed also methodological quality of the single included studies, but the result was not discussed. Thus, an analysis of data from studies of poor quality may give readers misleading information about good evidence in regard to an exercise in spite of the quality of the SR being high based on the AMSTAR criteria.

Based on the low AMSTAR score in Roberts et al. [30], few pooled analyses performed on other exercises than pursed-lip breathing in Holland et al. [14], and no SRs found on body position and relaxation exercises, additional RCT designs in BCEs are needed before conclusions on their efficacy can be made. In addition the variable results of the quality of the single studies included in the different SRs may be indicating a need for further research on BCEs and RMT.

In our overview, we found that, among the five SRs performing pooled statistics [13, 14, 31–33], only the high-quality SRs [14, 31, 33] performed a publication-biased analysis or mentioned the limitation of not performing publication- biased analyses. Publication-biased analysis involves a statistical test, such as Egger’s test, and/or a graphic aid, such as a funnel plot [16]. Egger’s test is a statistical test for funnel plot asymmetry. A possible asymmetry in a funnel plot might be due to selection bias, such as language bias, or poor methodological quality of the studies included in the SRs [35, 38]. Therefore, not applying the publication-biased criteria in the SRs might result in presenting more positive effects from the studies included. Although this criterion was fulfilled, these tests were sparsely discussed in the SRs.

BCEs and RMT are considered, used, and sometimes recommended as self-management techniques and training methods in the practice of pulmonary rehabilitation programs in the clinical care of patients with COPD [3, 10, 39, 40]. SRs on BCEs and RMT have become a popular and efficient way to summarise and synthesise research results as easily accessible knowledge for guiding clinical practitioners. This means that we need to ascertain how research results can best be organised and validated. When writing a SR, guided advice on how to perform a SR should be followed [11]. Our overview demonstrates that caution should be applied when interpreting the results of SRs. Authors of SRs should address and discuss the trustworthiness of information. In this way, authors and readers alike may arrive at a more-accurate picture of the evidence for BCEs and RMT.

### Discussion of the effect by performing breathing control exercises and respiratory muscle training

RMT and the various BCEs differ with respect to the reasons why they may reduce breathlessness. For instance, pursed-lip breathing may help individuals to increase of exhalation time resulting in a decrease in the respiratory rate and breathing more deeply. The exercise is often used spontaneously in severe stages of COPD [3]. Yoga breathing also focuses on deeper breathing but often in combination with relaxation and body positions [41]. Further, diaphragmatic breathing focuses on activating the diaphragm during inspiration and, at the same time, minimizing the actions of accessory muscles [26]. The diaphragm muscle may be more shortened in severe stages of COPD due to hyperinflation. Poor movement of the diaphragm muscle may thus be the reason for diaphragmatic breathing failing to improve breathlessness [26]. The single studies included in the high-quality SR by Holland et al. [14] evaluated patients with lung functioning between severe and moderate stages of COPD (i.e. FEV_1_ % 30-51%); thus, more severe COPD may be a reason for a lack of efficacy in some of the studies. Although there are differences, all of them focus on improving the breathing pattern and reducing breathlessness. The sensory cortex may contribute to the improved sensation of breathlessness [42].

The principle of inspiratory muscle training is to improve the strength of the diaphragm and the external intercostal muscles [3, 10]. Evidence shows that improvement of inspiratory muscle strength might be related to decreased effort in breathing and a positive change in the experience of breathlessness [43]. For instance, inspiratory muscle training may lead to a decrease of the inspiratory time, which leads to longer exhalation time, in turn leading to relaxation of the muscles [44]. The aim of this overview was to report subjective outcomes, but in relation to inspiratory muscle training. The improvement of inspiration muscle strength [31, 32, 34] and inspiratory muscle endurance [31, 34] shown in the different SRs (i.e. high and moderate quality) may explain the improvement in breathlessness. Higher training intensity and frequency were also reported or discussed as influencing a better outcome [32–34], but no clear concluding advice was given. Due to hyperinflation and a shortening of the diaphragm, the effects of inspiratory muscle training may differ with regard to the stages of COPD and different impairment of the respiratory muscles. Most of the SRs included studies with a mean predicted lung function <55% (Table 2), but few specified any differences in the severity of COPD when reporting improvement of breathlessness. The SR by Gosselink et al. [31] was the only one to evaluate the effects based on respiratory impairment at baseline (i.e. Pimax < 60 cm H_2_O), finding that these patients were more likely to have improved strength of the respiratory muscles. In the other SRs, there were no clear conclusions about which COPD patients might gain the most benefit from performing inspiratory muscle training [13, 32, 34]. Only one high-quality SR [34] combined inspiratory muscle training and expiratory muscle training in the meta-analysis. In fact, most of the studies included in the other SRs used only inspiratory muscle training. Furthermore, the studies in the pooled analysis used different inspiratory muscle-training techniques (see Figure 1 and Table 3). It is difficult, therefore, to provide detailed advice about which techniques are most beneficial in the practice of RMT. Hence, additional studies should focus on the effects of different methods of respiratory muscle-training techniques.

Although BCEs and RMT differ in their therapeutic approach and focus, the SRs found in our overview demonstrate that they have the similar goal to reduce breathlessness. Furthermore, BCEs and RMT require guidance by a respiratory health care professional who has the skills [9]. BCEs may be considered applied to patients with COPD with the aim to manage and control breathing during for instance exertion. Inspiratory muscle training, however, requires a training program based on respiratory muscle strengthening before possible improvement in breathlessness. In the high-quality SRs of inspiratory muscle training, interventions lasted from 3 to12 months with training duration of approximately 15 to 60 minutes 1 to 2 times a day for 5 to 7 days per week [31, 33], and learning the breathing-control exercises lasted from 4 to 12 weeks [14]. However, compliance with the practices of BCEs and RMT in a home situation may be difficult and may be a reason for biased results in the variable effects found especially in breathing control exercises.

It is not clear why there are effects of performing BCEs and RMT on other symptoms and QOL. We may hypothesise that an improvement in breathlessness may lead to improvement of other symptoms and QOL due to the close associations reported in the literature [5]. Most of the improvements in QOL were in disease-related QOL [13, 14, 31]. Disease-specific QOL often involves disease-related symptoms such as breathlessness [18] and may also be a reason for effects seen in the high-quality SRs on breathing control exercises [14] and respiratory muscle training [31, 33].

### Limitations

An overview of systematic reviews has several limitations. Detailed information from the single studies in the different SRs are, for instance, not presented and discussed. Furthermore, we have not been able to pool data from the different SRs. Because several of the same single studies were included in different SRs, we concluded that pooled data analyses and comparison of results between them would not be correct. Here we can mention that Gosselink et al. [31] and O’Brian et al. [32] include several of the same studies, using different concepts for the control groups (i.e. sham training alone). Another limitation is due to our focus on subjective outcome in this overview, which is the reason why we have not reported and discussed in detail physical outcomes or which of the RMTs are most efficient.

## Conclusion

Seven SRs have been conducted on breathing control exercises and respiratory muscle training in patients with COPD. According to the AMSTAR criteria, three were of high quality (two on respiratory muscle training and one on pursed-lip breathing, diaphragmatic breathing, yoga breathing), three were of moderate quality (respiratory muscle training), and one was of low quality (pursed-lip breathing). In the high-quality SRs, positive effects of performing inspiratory muscle training on breathlessness and QOL as well as on fatigue were found in one SR. Also one high-quality SR reported a positive effect on breathlessness of performing pursed-lip breathing. According to the authors of the SRs, the single RCTs included were of variable quality, indicating that more studies are needed. In the low-quality SR and the moderate-quality SRs, it has been difficult to fulfil the AMSTAR criteria, due partly, for instance, to the small number of RCT-based studies, not including all languages, and not performing publication-biased analysis. Recommended guidelines for writing an SR should be followed in order to provide high-quality SRs. Our overview demonstrates the need for more studies using the RCT design, especially on breathing control exercises but also on the different techniques of respiratory muscle training, before conclusive high-quality SRs can be performed.

## Electronic supplementary material

Additional file 1:**Search strategy.**(DOC 114 KB)
