# Supplementary material for: Effects of controlled breathing exercises and respiratory muscle training in people with chronic obstructive pulmonary disease: results from evaluating the quality of evidence in systematic reviews
Source: BMC Pulm Med. 2014 Nov 21;14:184. doi: 10.1186/1471-2466-14-184 (PMC4258938; doi:10.1186/1471-2466-14-184)
Supplement: Supplementary file 1 — Additional file 1:Search strategy.(DOC 114 KB) [file 12890_2013_618_MOESM1_ESM.doc]

| Number of articles in database | | | | | | | | | | |
| --- | --- | --- | --- | --- | --- | --- | --- | --- | --- | --- |
| Search number | Key word or MeSh term and combinations | PubMed (major) | Ovid (focus) | Cinahl (major) | PsycInfo | Amed (exp) | Cochrane | PEDro | | N |
| 1 | Lung disease | 549471 | 43758 | 2934 | 497# | 4418 | 26546 |  | |  |
| 2 | Lung disease, obstructive | 134001 | 13187 | 1630 | 0# | 0# | 13554 |  | |  |
| 3 | Pulmonary disease, chronic obstructive | 27356 | 17971 | 5933 | 0# | 6 | 2349 |  | |  |
| 4 | Pulmonary emphysema | 9493 | 9369 | 711## | 81# | 2804 | 206 |  | |  |
| 5 | Bronchitis chronic | 539 | 545 | 161 | 6 | 7# | 79 |  | |  |
| 6 | Pharmacology | 89619 |  |  |  |  |  |  | |  |
| 7 | Oxygen therapy | 12775 |  |  |  |  |  |  | |  |
| 8 | Respiration | 51613 | 37413 | 1569 | 7705# | 1645 | 5551 |  | |  |
| 9 | Breathing exercises | 1566 | 1570 | 540 | 178# | 230 | 500 |  | |  |
| 10 | Yoga | 1104 | 1114 | 1104 | 1624# | 393 | 279 |  | |  |
| 11 | Pranayama | 140# | 140# | 32 | 44# | 28# | 64# |  | |  |
| 12 | Mind–body therapies | 24406 | 345 | 664### | 67# | 41# | 4046 |  | |  |
| 13 | Muscle stretching exercises | 587 | 598 | 7 | 4# | 103 | 239 |  | |  |
| 14 | Relaxation | 4175 | 719 | 552 | 13760# | 974 | 1101 |  | |  |
| 15 | Breathing control exercises | 3779# | 3 | 0 | 0# | 0# | 1838# |  | |  |
| 16 | Diaphragmatic breathing | 5799# | 144 | 64# | 112# | 32# | 184# |  | |  |
| 17 | Purse lip breathing | 51# | 38 | 19# | 2# | 9# | 44# |  | |  |
|  |  |  |  |  |  |  |  |  | |  |
| 18 | (1–5) OR | 579471 | 83981 | 11252 | 577 | 4421 | 26546 |  | |  |
| 19 | 18 NOT (6–7) | 545931* |  |  |  |  |  |  | |  |
| 20 | 18 AND 8 | 6714* | 1413 | 92 | 36 | 300 | 1570 |  | |  |
| 21 | 18 AND 9 | 485* | 293 | 83 | 1 | 77 | 188 |  | |  |
| 22 | 18 AND 10 | 45* | 9 | 6 | 0 | 13 | 21 |  | |  |
| 23 | 18 AND 11 | 12* | 3 | 2 | 0 | 4 | 8 |  | |  |
| 24 | 18 AND 12 | 735* | 0 | 1 | 0 | 2 | 264 |  | |  |
| 25 | 18 AND 13 | 3* | 3 | 0 | 0 | 0 | 2 |  | |  |
| 26 | 18 AND 14 | 121* | 1 | 2 | 9 | 15 | 35 |  | |  |
| 37 | 18 AND 15 | 743* | 2 | 0 | 0 | 0 | 495 |  | |  |
| 38 | 18 AND 16 | 748* | 33 | 12 | 1 | 7 | 47 |  | |  |
| 29 | 18 AND 17 | 37* | 26 | 120 | 0 | 6 | 19 |  | |  |
| 30 | (20–29) OR | 8136 | 1725 | 180 | 45 | 401 | 2070 |  | |  |
| 31 | Differences in limitations of each database | Limitation: Human, review, Date: Jan 1..2002–December.31.2013 | Limitation: Human, Review, Date: 2002–2013 | Limitation: Systematic review, Human  Date: Jan 1. 2002–December 31.2013 | Limitation: Date: 2002–2013 | Limitation: Review Date: 2002–2013 | Limitation: Cochrane reviews and others from 2002-2013 | Search words: Chronic Obstructive Pulmonary Disease AND Review  Date: From 2002-2013 | |  |
| 32 | Number of articles after limitation | 317 | 40 | 5 | 25 | 3 | 138 | 114 | | 642 |
| 33 | Number of articles after sorting out the same article in different database |  |  |  |  |  |  |  | | 548 |
| 34 | Number of articles after hand search of inclusion and exclusion criteria from titles |  |  |  |  |  |  |  | | 111 |
| 35 | Result |  | | | | | | | 7 | |

*Search words combination 18 is search words combination 19

# Not a MeSh term or key word in the database, ## Search term “Emphysema”, ### search term “Mind Body Techniques”
